# Polymorphisms in CXCR3 ligands predict early CXCL9 recovery and severe chronic GVHD

**DOI:** 10.1038/s41408-021-00434-2

**Published:** 2021-02-27

**Authors:** Hao Dai, Sivaramakrishna P. Rachakonda, Olaf Penack, Igor W. Blau, Olga Blau, Aleksandar Radujkovic, Carsten Müller-Tidow, Peter Dreger, Rajiv Kumar, Thomas Luft

**Affiliations:** 1 Department of Epidemiology, German Cancer Research Centre (DKFZ), Heidelberg, Germany; 2 Division of Hematology, Oncology and Tumorimmunology, Charité University Medicine Berlin, Berlin, Germany; 3 Department of Medicine V, University Hospital Heidelberg, Heidelberg, Germany

**Keywords:** Risk factors, Translational research

## Abstract

Chronic graft-versus-host disease (cGVHD) is a major cause of mortality and morbidity after allogeneic stem cell transplantation (alloSCT). The individual risk of severe cGVHD remains difficult to predict and may involve CXCR3 ligands. This study investigated the role of single-nucleotide polymorphisms (SNPs) of CXCL4, CXCL9, CXCL10, and CXCL11, and their day +28 serum levels, in cGVHD pathogenesis. Eighteen CXCR3 and CXCL4, CXCL9–11 SNPs as well as peri-transplant CXCL9–11 serum levels were analyzed in 688 patients without (training cohort; *n* = 287) or with statin-based endothelial protection cohort (*n* = 401). Clinical outcomes were correlated to serum levels and SNP status. Significant polymorphisms were further analyzed by luciferase reporter assays. Findings were validated in an independent cohort (*n* = 202). A combined genetic risk comprising four CXCR3 ligand SNPs was significantly associated with increased risk of severe cGVHD in both training cohort (hazard ratio (HR) 2.48, 95% confidence interval (CI) 1.33–4.64, *P* = 0.004) and validation cohort (HR 2.95, 95% CI 1.56–5.58, *P* = 0.001). In reporter assays, significantly reduced suppressive effects of calcineurin inhibitors in constructs with variant alleles of rs884304 (*P* < 0.001) and rs884004 (*P* < 0.001) were observed. CXCL9 serum levels at day +28 after alloSCT correlated with both genetic risk and risk of severe cGVHD (HR 1.38, 95% CI 1.10–1.73, *P* = 0.006). This study identifies patients with high genetic risk to develop severe cGVHD.

## Introduction

Chronic graft-versus-host disease (cGVHD) is a leading cause of morbidity and mortality after allogeneic stem cell transplantation (alloSCT)^1–3^. cGVHD encompasses dysregulated immune responses, chronic inflammation, and fibrosis, with various manifestations resembling autoimmune diseases^4^. Steroid-based immunosuppressive therapy achieves meaningful responses in only 50% of the patients^5,6^. cGVHD can be graded as severe or non-severe cGVHD according to the National Institutes of Health (NIH) consensus criteria^7^. Non-severe cGVHD is associated with better overall survival because of an increased graft-versus-leukemia (GVL) effect, while severe cGVHD causes significant morbidity and adverse impact on survival in long-term survivors^8^. Therefore, better prediction algorithms of severe cGVHD might improve risk-benefit assessments for patients before undergoing alloSCT.

Accumulating evidence suggests that type 1 T helper (Th1) cells play a critical role in the pathogenesis of cGVHD^9–11^. CXCR3 is a Th1-associated inflammatory chemokine receptor highly expressed on effector T cells, in particular Th1 cells^12^. CXCR3 ligands include interferon-γ (IFN-γ)-inducible chemokines of the CXC family such as CXCL9, CXCL10, and CXCL11^13–15^, as well as CXCL4 (PF4) and CXCL4V1 (PF4V1)^16,17^. CXCR3^+^ Th1 can increase local secretion of IFN-γ in inflamed tissues, which in turn stimulates CXCR3 ligand expression. In pathological conditions, the interplay between CXCR3 pathway and IFN-γ can lead to excessive immune responses and amplified inflammation^18^. CXCR3 overexpression was also found in a B cell subset that is expanded in cGVHD patients^19^. Furthermore, the CXCR3 axis has been shown to regulate endothelial cell apoptotic death, while endothelial cell alteration is a typical finding in cGVHD^20,21^. Increased expression of CXCR3 ligands was found in both target tissues and the circulatory system in cGVHD^22–27^. Thus, CXCR3 ligands are potentially important mediators of cGVHD.

In the present study, we investigated the interplay of serum CXCR3 ligand concentrations and selected polymorphisms with regard to the incidence of severe cGVHD after alloSCT.

## Patients and methods

### Patient cohorts

Patients were recruited from two independent transplant centers and divided into three cohorts, including the training cohort (without statin-based endothelial protection (SEP), Heidelberg), SEP cohort (Heidelberg), and validation cohort (no SEP, Berlin). Patients were eligible if they had been transplanted between 2002 and 2014 in one of the two institutions, survived at least 6 months after alloSCT, and had biological material as needed for this study available. Human leukocyte antigen (HLA)-identical related donors were chosen if an HLA-identical (10/10) unrelated donor was not available or if an HLA-matched unrelated donor (9/10) was not available. SEP consists of pravastatin 20 mg/day and ursodeoxycholic acid (UDCA) 3 × 250 mg/day^28,29^, and was used as a standard treatment in Heidelberg University Hospital since 01/2010. Written informed consent according to the Declaration of Helsinki was obtained from all patients, and the local Ethics committees had approved sample and data collection.

### GVHD prophylaxis and cGVHD diagnosis and grading

GVHD prophylaxis and treatment were performed as previously described^30^. Severe cGVHD was defined as previously described according to the NIH consensus criteria^7^, namely involvement of at least one organ system in its severest form (corresponding to being awarded 3 points in the NIH grading system, with the exception of lung cGVHD for which 2 points are sufficient). Non-severe cGVHD was defined as any cGVHD not fulfilling the criteria of severe cGVHD^8^.

### Single-nucleotide polymorphism (SNP) analyses

A total of 18 polymorphisms from *CXCL4*, *CXCL4V1*, *CXCL9–11*, and *CXCR3* genes were identified by tagging approach using 1000 Genomes database for the Caucasian population. For *CXCL4* and *CXCL4V1* locus, a region of 152 kb (hg19: Chr4: 74,708,750–74,861,174) was screened and seven SNPs (rs409336, rs6810940, rs655328, rs28472816, rs3097412, rs1429638, and rs17811212; Supplemental Fig. 1) with minor allele frequency ≥0.05 that tagged an additional 147 SNPs (*r*^2^ ≥ 0.8, 71% of SNPs in the region) were selected. For *CXCL9–11* locus, a region of 200 kb (hg19: Chr4: 76,842,428–77,042,568) was screened and seven SNPs (rs8878, rs884304, rs3733236, rs2276885, rs6849878, rs67413521, and rs4282209; Supplemental Fig. 1) with minor allele frequency ≥0.05 that tagged an additional 456 SNPs (*r*^2^ ≥ 0.8, 76% of SNPs in the region) were selected. For *CXCR3* gene locus, a region of 43 kb (hg19: ChrX: 70,815,766–70,858,367) was screened and four SNPs (rs2280964, rs3091304, rs6625809, and rs3091305; Supplemental Fig. 1) with minor allele frequency ≥0.05 that tagged an additional two SNPs (*r*^2^ ≥ 0.8) in the region were selected for genotyping. Genomic DNA from patients was genotyped using an allele-specific method (TaqMan Technology, Applied Biosystems, Foster City, CA). For quality control purpose, 10% of the samples was blindly replicated. PCR plates were read on a ViiA7 real-time instrument (Applied Biosystems) and QuantStudio Real-Time PCR Software was used to call genotypes.

### Serological analyses

Prospectively collected serum samples taken once weekly before alloSCT until day +28 were analyzed for CXCL9 serum levels. Kinetics of CXCL9 levels were assessed in the context of calcineurin inhibitors (CNIs). For this purpose, a slope (differences/time) was calculated representing the recovery rate of serum CXCL9 until day +28 $({\mathrm{Slope}}\;{\mathrm{CXCL}}9 = \frac{{{\mathrm{CXCL}}9_{{\mathrm{day}}\;28} - {\mathrm{Lowest}}\;{\mathrm{CXCL}}9_{{\mathrm{days}}\;0 - 14}}}{{28 - {\mathrm{day}}_{{\mathrm{lowest}}\;{\mathrm{CXCL}}9}}})$. Serum CXCL10 and CXCL11 levels were analyzed before transplant and on day +28. Endothelial markers angiopoietin-2 and sCD141 on day +28 were measured by ELISA (R&D, UK).

### Dual-luciferase reporter assays

The *CXCL9* promoter region with 6.8 kb (hg19, Chr4: 76,928,615–76,935,456) upstream of the start codon was cloned into pGL4.10 [luc2] promoter-less vector (“wild-type construct”). In addition, we generated four reporter constructs with QuikChange Site-Directed Mutagenesis Kit (Invitrogen). Three constructs carried variant alleles for rs884304 and two tagged SNPs (rs884004 and rs2869462) and one construct carried variant alleles for all the three SNPs. All five constructs were confirmed by Sanger sequencing. The constructs were transiently transfected into HEK293T cells together with Renilla luciferase vector as control using Lipofectamine 2000 reagent (Invitrogen) and incubated for 24 h without treatment or treated with IFN-γ (300 U/mL) and/or cyclosporin A (CsA, 1 μM) or FK506 (tacrolimus, 1 μM). Cells were subsequently lysed and assayed for luciferase activity. The effect of CNIs on *CXCL9* promoter activity of the constructs was calculated as:

$${\mathrm{Effect}}\;{\mathrm{of}}\;{\mathrm{CNIs}} = 1 - \frac{{{\mathrm{Luciferase}}\;{\mathrm{activity}}\;{\mathrm{with}}\;{\mathrm{CNIs}}}}{{{\mathrm{Luciferase}}\;{\mathrm{activity}}\;{\mathrm{without}}\;{\mathrm{CNIs}}}}$$

### Statistical analysis

Differences in categorical clinical covariates in patients were analyzed by Fisher’s exact test or *X*^2^ test. Mann–Whitney *U* test was used for comparisons of quantitative continuous variables. Genotype frequencies of SNPs were tested in subjects for deviation from Hardy–Weinberg equilibrium. Median follow-up time was estimated by reverse Kaplan–Meier estimator. CXCL9–11 serum levels were log 2-transformed because the distribution was highly left-skewed. Cause-specific Cox regression was used in univariate analysis, including CXCL9 serum levels as a continuous variable. The effect of the polymorphisms, using additive, dominant, and recessive models, was evaluated with regard to the risk of severe cGVHD, which was calculated from the date of alloSCT to the day of developing severe cGVHD. Relapse before severe cGVHD and non-relapse death without severe cGVHD were considered as competing events for severe cGVHD. To account for the competing risks, cumulative incidence function was implemented, and statistical significance was determined using the Gray’s *K*-sample test.

Severe cGVHD, relapse before severe cGVHD and NRM were treated as competing events and analyzed by cause-specific Cox regression models. Covariates included in multivariable models were age, diagnosis (lymphoid vs. myeloid), matched vs. mismatched donor, sex of donor and recipient, and usage of antithymocyte globulin (ATG). Prediction error curves and concordance index curves were generated to assess the model performance.

All statistical tests were two-sided. Hazard ratio (HR) was estimated with 95% confidence intervals (CIs). Values of *P* < 0.05 were considered statistically significant. All statistical analyses were carried out using statistical software R (version 3.5.2) together with the R packages dplyr (0.8.3), survival (2.44-1.1), cmprsk (2.2–8), and pec (2018.07.26).

## Results

### Patient characteristics

Overall, 688 patients (training cohort: *n* = 287; SEP cohort: *n* = 401) from Heidelberg University Hospital fulfilled the eligibility criteria for this study (Supplemental Fig. 2). Of these, 545 patients (no SEP: *n*=242; SEP: *n*=303) with available DNA were successfully genotyped for 4 *CXCR3* and 14 *CXCL4/4V/9/-10/-11* polymorphisms. Two hundred and two patients from Charite (Berlin) qualified for the validation cohort. Baseline and transplant-associated characteristics are shown in Table 1 and Supplemental Table 1. Overall, 50 of 287 patients (17%) in the training cohort, 53 of 401 patients (13%) in the SEP cohort, and 48 of 202 patients (24%) in the validation cohort developed at least one episode of severe GVHD after a median time of 12.9, 12.5, and 13.8 months, respectively. The estimated median follow-up time was 72.5 months (95% CI 67.1–77.3) for patients in Heidelberg center and 66.7 months (95% CI 60.6–77.3) for Berlin cohort.

**Table 1:** Patient characteristics in the training cohort, validation cohort, and SEP cohort.

	Training cohort no SEP, *n* = 287	Validation cohort no SEP, *n* = 202	SEP cohort, *n* = 401
Median age at alloSCT (years, range)	50 (17–71)	50 (19–72)	57 (19–76)
Sex (*n*, %)			
Female	114 (40)	72 (36)	161 (40)
Male	173 (60)	130 (64)	240 (60)
Donor			
RD	108 (38)	65 (32)	104 (26)
UD	179 (62)	137 (68)	297 (74)
Donor–recipient HLA matching			
Matched donor	214 (75)	195 (97)	327 (82)
Mismatched dono	73 (25)	7 (3)	74 (18)
Sex mismatch (donor–recipient) (*n*, %)			
Male–male, Female–Female	164 (57)	109 (54)	222 (55)
Male–female	71 (24)	45 (22)	103 (26)
Female–male	52 (19)	48 (24)	76 (19)
Disease (*n*, %)			
AML	76 (26)	83 (41)	146 (36)
MDS, MPN, AA	45 (16)	44 (22)	72 (18)
Lymphoma, ALL, CLL	111 (39)	61 (30)	141 (35)
MM, Amyloidosis	55 (19)	14 (7)	42 (11)
Disease score before alloSCT (*n*, %)			
0	99 (34)	92 (46)	138 (34)
1	41 (15)	80 (40)	147 (37)
2	134 (47)	25 (12)	108 (27)
NA	13 (5)	5 (2)	8 (2)
Stem cell source (*n*, %)			
Peripheral stem cells	272 (95)	200 (99)	382 (95)
Bone marrow stem cells	15 (8)	2 (1)	19 (5)
Conditioning (*n*, %)			
MAC	51 (18)	82 (41)	15 (4)
APL	7 (2)		46 (11)
RIC	229 (80)	120 (59)	340 (85)
ATG (*n*, %)			
No	140 (49)	81 (40)	103 (26)
Yes	147 (51)	121 (60)	298 (74)
Combined genetic score (*n*, %)			
Low-risk	190 (66)	160 (79)	235 (59)
High risk	52 (18)	42 (21)	68 (17)
NA (no DNA)	45 (16)		98 (24)
CXCL9 pre^a^ median (pg/mL, IQR)	*n* = 109 204.5 (108.7–629.5)		*n* = 296 175.7 (72.3–542.1)
CXCL9 day 28+^b^ median (pg/mL, IQR)	*n* = 152 315.3 (141.2–673.3)		*n* = 342 295.5 (114.8–598.8)
CXCL10 pre median (pg/mL, IQR)			
CXCL10 day 28+ median (pg/mL, IQR)	*n* = 146 152.9 (65.6–283.7)		*n* = 333 128.7 (56.7–242.0)
CXCL11 pre median (pg/mL, IQR)	*n* = 103 79.8 (24.3–183.4)		*n* = 289 70.4 (24.5–165.0)
CXCL11 day 28+ median (pg/mL, IQR)	*n* = 146 119.2 (61.2–211.8)		*n* = 333 122.1 (53.5–204.6)

*SEP* statin-based endothelial prophylaxis, *AA* aplastic anemia, *ALL* acute lymphoblastic leukemia, *alloSCT* allogeneic stem cell transplantation, *AML* acute myelogenous leukemia, *ATG* antithymocyte globulin, *CLL* chronic lymphocytic leukemia, disease score: 0 = CR1, 1 = CR2, 2 = all other, *MAC* myeloablative conditioning, *MPS* myeloproliferative syndrome, *MM* multiple myeloma, *RD* related donor, *RIC* reduced intensity conditioning, *UD* unrelated donor.

^a^Measured pre-transplant.

^b^Measured at day 28 post transplant.

### Identification of high-risk genotypes of SNPs from *CXCR3* and ligand genes (*CXCL4/9–11*) in the training cohort

**Fig. 1:**
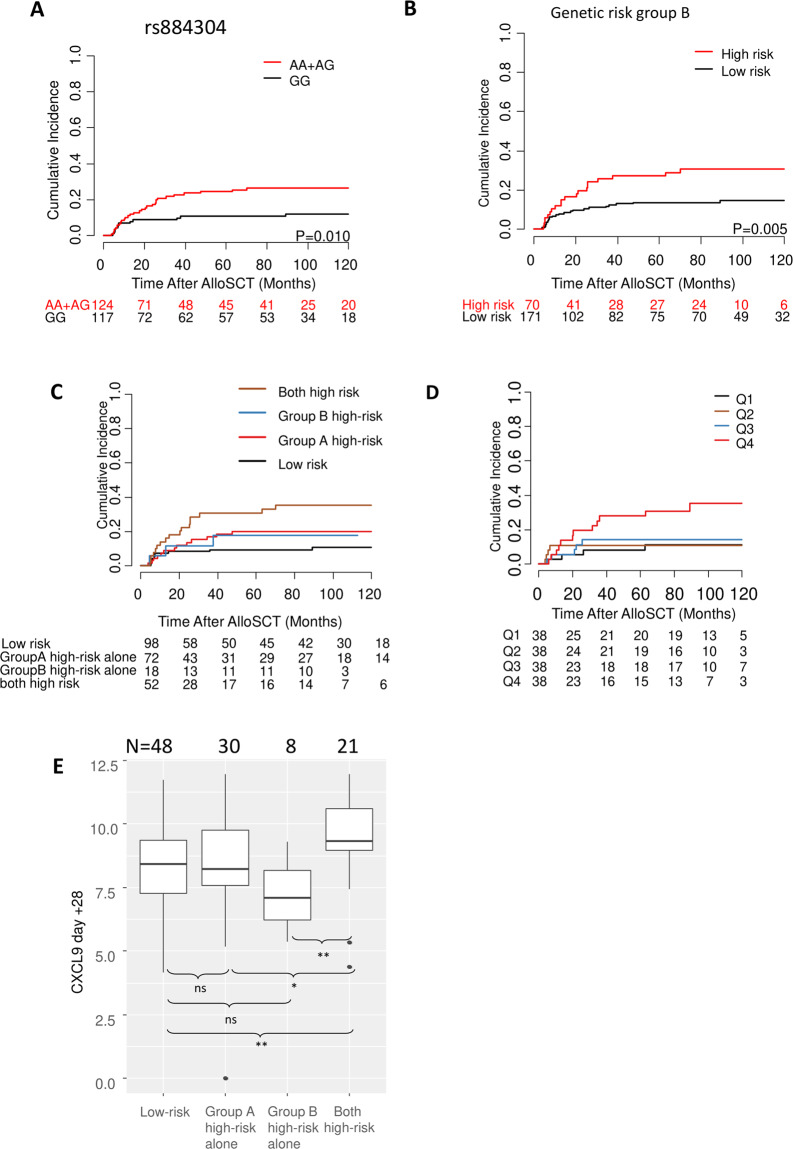
Association of single-nucleotide polymorphisms (SNPs) in *CXCR3* ligand genes and serum chemokines with the incidence of severe chronic graft-versus-host disease (GVHD). **A** Carriers of the variant allele for *CXCL9* polymorphism rs884304 (AA + AG) were associated with a significantly higher incidence of severe chronic GVHD in the training cohort. **B** Effects of the three candidate SNPs for genetic risk group B on the incidence of severe chronic GVHD after alloSCT. Genetic risk group B was based on rs3733236, rs4282209, and rs655328. **C** Association of severe chronic GVHD incidence with genetic risk groups A and B. **D** Association of serum CXCL9 levels with a cumulative incidence of severe chronic GVHD after alloSCT in the training cohort. **E** Association of day +28 CXCL9 serum levels with genetic risk groups A and B. n.s. not significant; *P ≤ 0.05; **P < 0.01; ***P < 0.001.

To determine the effect of polymorphisms on severe cGVHD, the polymorphisms were analyzed under the additive, dominant, and recessive models; the most significant statistic among the three models is considered to be an indicator of association between the polymorphism and the outcome (Supplemental Table 2). Out of the 18 polymorphisms analyzed, rs884304 in *CXCL9* locus showed statistically significant association (*P* = 0.010) with a risk of severe cGVHD in the dominant model. Patients who were carriers of the variant allele (AA/AG genotypes) of rs884304 showed significantly increased risk for severe cGVHD (HR 2.35, 95% CI 1.23–4.51) compared to patients with homozygote GG genotype (Fig. 1A). The other polymorphisms did not show any isolated statistically significant association with severe cGVHD in the training cohort.

### Selection of polymorphisms for computing genetic risk group

Two genetic risk groups A and B were built based on statistically significant polymorphism rs884304 and three other polymorphisms (rs3733236 (*CXCL9–11*), rs4282209 (*CXCL9–11*), rs655328 (*CXCL4V1*)) that showed a Gray’s test *P* value of < 0.10 with a risk of severe cGVHD (Supplemental Fig. 3).

For genetic risk group A, patients with high-risk genotypes (AA/AG) for rs884304 were marked as high-risk group, others (GG) were considered as low-risk group. For genetic risk group B, patients with rs3733236GG, rs4282209GG, and rs655328CC/CT at the same time were considered as high-risk group, others were classified as low-risk group. Besides genetic risk group A, genetic risk group B is also significantly associated with the risk of severe cGVHD (Fig. 1B, *P* = 0.005). If the two genetic risk groups A and B were assessed together, patients who were at high risk for both genetic risk groups were associated with the highest risk of severe cGVHD (Fig. 1C). Therefore, a combined genetic risk group was established for CXCR3 ligand genes (*CXCL4* and *CXCL9–11*). Patients with any low-risk genotypes for the two genetic risk groups (rs884304GG and three SNPs (rs3733236AA/AG, rs4282209AA/AG, and rs655328TT)) were classified into the low-risk group, patients with all high-risk genotypes (rs884304AA/AG, rs3733236GG, rs4282209GG, and rs655328CC/CT) were classified into high-risk group.

### Association of CXCL9 serum levels with risk of severe cGVHD

We previously demonstrated that CXCL9 levels correlate with the risk of developing severe cGVHD when measured at the onset of mild symptoms^8^. In the present study, we analyzed CXCL9, CXCL10, and CXCL11 serum levels before and on day +28 after alloSCT in the training cohort. In four increasing intervals (Q1, Q2, Q3, and Q4) separated by quartiles of CXCL9, patients in Q4 were associated with increased risk for severe cGVHD in the training cohort (Fig. 1D, Q4 vs. Q1–3, HR 2.93, 95% CI 1.34–6.43, *P* = 0.007). In univariable analysis, day +28 serum CXCL9 levels were significantly associated with risk of severe cGVHD in the training cohort (HR for a twofold change 1.38, 95% CI 1.10–1.73, *P* = 0.006).

No significant association was found for either CXCL10 (*P* = 0.176) or CXCL11 (*P* = 0.524) day +28 serum levels with risk of severe cGVHD. Similarly, serum levels of CXCL9, 10, and 11 taken prior to conditioning therapy did not correlate with severe cGVHD (*P* = 0.777, 0.805, and 0.346, respectively).

Comparing the genotype data with serum CXCL9 levels, patients in the combined genetic high-risk group had statistically significantly higher serum CXCL9 levels on day +28 post alloSCT (Fig. 1E).

### Combined genetic risk in the training and validation cohorts

**Fig. 2:**
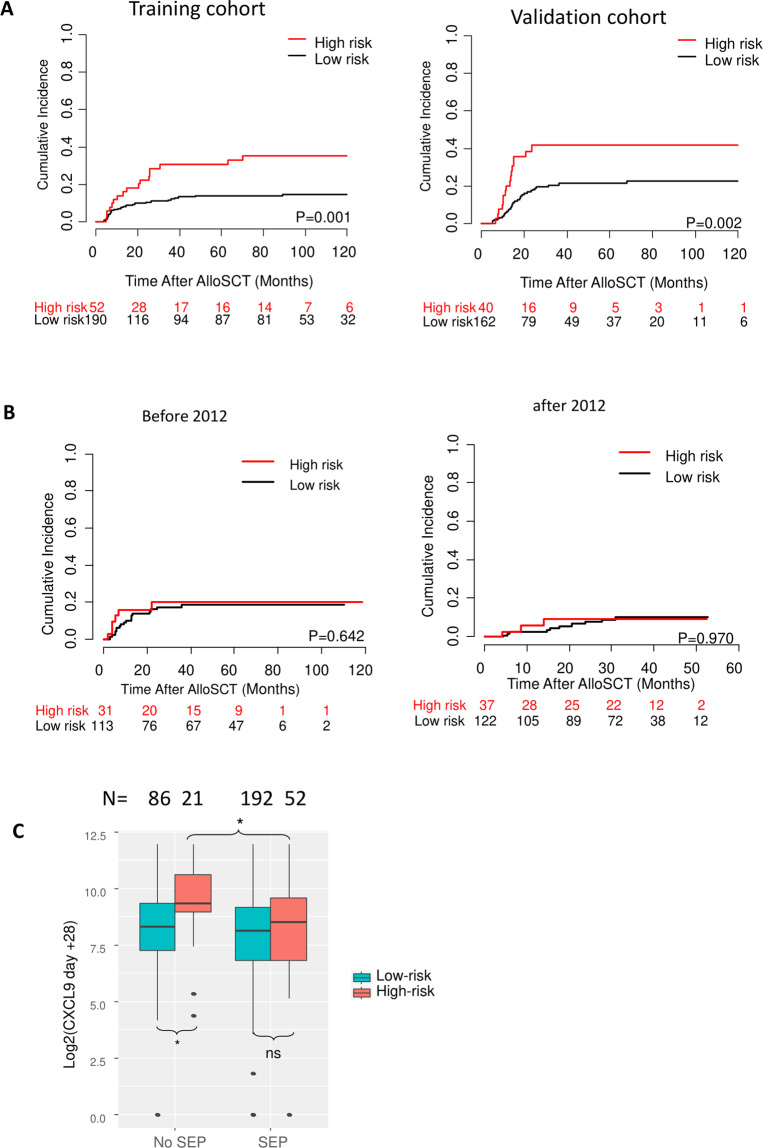
The effect of genetic risk (CXCR3 ligands) on severe chronic graft-versus-host disease (GVHD). **A** Association of the combined genetic risk group with the incidence of severe chronic GVHD in the training cohort and validation cohort. **B** No difference could be observed between the genetic risk groups in patients with SEP in two consecutive cohorts (*n* = 144 and 159, respectively). **C** Association of serum CXCL9 levels at day +28 with the combined genetic risk group in patients without and with SEP. n.s. not significant; *P ≤ 0.05.

Taken together, high-risk polymorphisms were found in 21% (52/242) patients in the training cohort and 20% (40/202) patients in the validation cohort (Table 1). The high-risk genotype was significantly associated with an increased risk of severe cGVHD in both the training and validation cohort (Fig. 2A).

Multivariable analyses to assess the influence of high-risk genotypes on outcomes after alloSCT were performed with covariates age, diagnosis (lymphoid vs. myeloid), matched vs. mismatched donor, sex of donor and recipient, and usage of ATG. As this model only comprised pre-transplantation factors, serum levels of day +28 CXCL9 were not included. Results are shown in Tables 2 and 3. For severe cGVHD, similar HRs for the high-risk group were observed in the training (HR 2.48, 95% CI 1.33–4.64, *P* = 0.004) and the validation cohort (HR 2.95, 95% CI 1.56–5.58, *P* = 0.001).

**Table 2:** Multivariable analysis of the incidence of severe cGVHD, OS, NRM, and relapse in the training cohort.

Covariates	Severe chronic GVHD^a^				OS				NRM^a^		Relapse^a^	
	*N*	ScGVHD	HR (95% CI)	*P*	*N*	death	HR (95% CI)	*P*	HR (95% CI)	*P*	HR (95% CI)	*P*
Genetic risk												
Low-risk group	190	26	Ref.		190	97	Ref.		Ref.		Ref.	
High-risk group	52	17	2.48 (1.33–4.64)	0.004	52	27	1.09 (0.70–1.69)	0.697	1.34 (0.68–2.65)	0.402	0.90 (0.52–1.57)	0.710
Age	242	43	1.01 (0.98–1.03)	0.622	242	124	1.02 (1.00–1.03)	0.041	1.04 (1.01–1.07)	0.006	1.00 (0.98–1.02)	0.754
Recipient sex												
Female	93	19	Ref.		93	48	Ref.		Ref.		Ref.	
Male	149	24	0.88 (0.47–1.64)	0.694	149	76	1.00 (0.69–1.45)	0.979	1.52 (0.80–2.90)	0.199	0.77 (0.49–1.21)	0.252
Donor sex												
Female	74	21	Ref.		74	38	Ref.		Ref.		Ref.	
Male	168	22	0.5 (0.29–0.98)	0.042	168	86	1.07 (0.72–1.58)	0.741	0.95 (0.50–1.78)	0.863	0.93 (0.57–1.49)	0.755
Donor												
Matched donor	179	34	Ref.		179	91	Ref.		Ref.		Ref.	
Mismatched donor	63	9	1.82 (0.76–4.08)	0.180	63	33	1.45 (0.91–2.32)	0.115	2.33 (1.08–5.03)	0.032	0.92 (0.51–1.66)	0.772
Disease type												
Myeloid^b^	102	20	Ref.		102	47	Ref.		Ref.		Ref.	
Lymphoid^c^	140	23	1.05 (0.57–1.95)	0.869	140	77	1.25 (0.86–1.82)	0.238	0.78 (0.43–1.43)	0.419	2.26 (1.38–3.71)	0.001
ATG												
No	117	32	Ref.		117	68	Ref.		Ref.		Ref.	
Yes	125	11	0.26 (0.11–0.59)	0.001	125	56	0.68 (0.45–1.03)	0.067	0.49 (0.24–1.02)	0.057	0.86 (0.52–1.42)	0.559

*ATG* antithymocyte globulin, *CI* confidence interval, *HR* hazard ratio, *ScGVHD* severe chronic graft-versus-host disease.

^a^HR and *P* value from cause-specific Cox models for competing risk. Death without scGVHD was taken as the competing event for scGVHD. NRM and relapse were treated as competing events.

^b^Myeloid: acute myeloid leukemia and myelodysplastic and myeloproliferative syndromes.

^c^Lymphoid: acute lymphoblastic leukemia, chronic lymphocytic leukemia, T/B cell lymphoma, and multiple myeloma.

**Table 3:** Multivariable analysis of the incidence of severe cGVHD, OS, NRM, and relapse in the validation cohort.

Covariates	Severe chronic GVHD^a^				OS				NRM^a^		Relapse^a^	
	*N*	ScGVHD	HR (95% CI)	*P*	*N*	death	HR (95% CI)	*P*	HR (95% CI)	*P*	HR (95% CI)	*P*
Genetic risk												
Low-risk group	162	32	Ref.		162	66	Ref.		Ref.		Ref.	
High-risk group	40	16	2.95 (1.56–5.58)	0.001	40	17	1.16 (0.67–2.00)	0.590	1.74 (0.68–4.43)	0.248	0.78 (0.42–1.44)	0.423
Age	202	48	1.00 (0.98–1.02)	0.970	202	83	1.01 (0.99–1.03)	0.278	1.03 (0.99–1.06)	0.176	1.01 (0.99–1.03)	0.331
Recipient sex												
Female	72	20	Ref.		72	29	Ref.		Ref.		Ref.	
Male	132	28	0.69 (0.38–1.26)	0.225	132	54	0.92 (0.58–1.47)	0.737	0.41 (0.17–1.00)	0.050	1.01 (0.63–1.64)	0.957
Donor sex												
Female	75	23	Ref.		75	29	Ref.		Ref.		Ref.	
Male	127	25	0.75 (0.41–1.37)	0.344	127	54	1.40 (0.87–2.26)	0.160	2.14 (0.83–5.53)	0.117	1.24 (0.76–2.04)	0.391
Donor												
Matched donor	195	47	Ref.		195	79	Ref.		Ref.		Ref.	
Mismatched donor	7	1	0.98 (0.13–7.64)	0.984	7	4	2.55 (0.90–7.19)	0.078	6.46 (1.33–33.16)	0.020	1.14 (0.27–4.74)	0.859
Disease type												
Myeloid^b^	127	29	Ref.		127	51	Ref.		Ref.		Ref.	
Lymphoid^c^	75	19	0.77 (0.41–1.44)	0.416	75	32	1.08 (0.67–1.73)	0.762	2.10 (0.84–5.28)	0.115	1.05 (0.64–1.74)	0.842
ATG												
No	81	29	Ref.		81	37	Ref.		Ref.		Ref.	
Yes	121	19	0.41 (0.22–0.76)	0.005	121	46	0.88 (0.55–1.41)	0.590	0.66 (0.26–1.64)	0.367	1.00 (0.61–1.65)	0.988

*ATG* antithymocyte globulin, *CI* confidence interval, *HR* hazard ratio, *scGVHD* severe chronic graft-versus-host disease.

^a^HR and *P* value from cause-specific Cox models for competing risk. Death without scGVHD was taken as the competing event for scGVHD. NRM and relapse were treated as competing events.

^b^Myeloid: acute myeloid leukemia and myelodysplastic and myeloproliferative syndromes.

^c^Lymphoid: acute lymphoblastic leukemia, chronic lymphocytic leukemia, T/B cell lymphoma, and multiple myeloma.

The result of the training cohort was validated using a cause-specific Cox model fitted to the validation cohort with an offset equal to the effect of the genetic risk group in the training cohort. The re-estimated effect of genetic risk group in the validation cohort was not significantly different from 1 (HR 1.19, 95% CI 0.63–2.24, *P* = 0.60). This means the effect of genetic risk group in the validation cohort was not significantly different from the effect in the training set. The effect was also validated using prediction error curves. A detailed validation method is provided in Supplemental statistical methods (Supplemental Fig. 4).

The predictive power of the genetic risk for severe cGVHD was assessed using prediction error and concordance index over time of two different cause-specific Cox models. The reference models included only the clinical parameters: age, diagnosis (lymphoid vs. myeloid), matched vs. mismatched donor, sex of donor and recipient, and usage of ATG. CXCL models included the genetic risk group (high-risk vs. low-risk) predictor in addition to the same clinical parameters. Lower prediction error and higher concordance index indicate better performance of a model. The model with the genetic risk group predictor obtained significantly lower prediction error and significantly higher concordance index over time in both training and validation cohort (Supplemental Fig. 5).

### Loss of predictive impact of the genetic risk group in the context of SEP

Patients receiving SEP were consecutively recruited upon standard policy change from 01/2010 in the Heidelberg center. In order to control for random effects due to alterations in clinical practice over time, patients with SEP were divided into two separate cohorts (patients transplanted before 12/2011, high risk: *n* = 31, low risk: *n* = 113; 01/2012–06/2014, high risk: *n* = 37, low risk: *n* = 122). The introduction of SEP abolished the impact of high-risk genotypes in both groups of patients (Fig. 2B). The protective effect of SEP was restricted to the high-risk group and the risk of severe cGVHD was reduced to the level of the low-risk group. The high-risk group also did not show statistically significant effects in the multivariable analysis (Supplemental Table 3; HR 1.48, 95% CI 0.68–3.21, *P* = 0.321). In addition, SEP was associated with significantly reduced serum CXCL9 levels at day +28. Again, the effect was confined to the genetic high-risk group (Fig. 2C).

### ATG reduced severe cGVHD risk irrespective of genetic risk

We investigated the effect of ATG on severe cGVHD and interaction with genetic risk groups. Patients receiving ATG had a significantly lower risk of developing severe cGVHD in both training and validation cohorts. Unlike SEP, the effect of ATG did not differ between genetic high- and low-risk groups of CXCR3 ligand genes (Supplemental Fig. 6), and within the ATG group, genetic high-risk patients still had an increased risk of severe cGVHD when compared to low-risk patients (combined training and validation cohorts, *n* = 474, HR 2.04, 95% CI 1.15–3.60, *P* = 0.015). In addition, ATG usage did not alter serum CXCL9 levels at day +28. In the high-risk group, SEP could fully substitute the protective effect of ATG, but there were no additive effects in patients receiving both. In contrast, ATG was effective independent of SEP in the low-risk group (Supplemental Fig. 7).

### The association of the combined genetic risk with acute GVHD

A prior episode of acute GVHD is a risk factor for cGVHD. We investigated the association of the high-risk genotype with acute GVHD and NRM after GVHD in the complete cohort of patients (including deaths before 6 months, Supplemental Table 4). No significant association was found for genetic high risk in the *CXCR3* ligand genes with the incidence of acute GVHD or grade 3–4 GVHD, nor with NRM after GVHD (Supplemental Table 5).

### Functional evaluation of *CXCL9* polymorphisms using dual-luciferase assays

**Fig. 3:**
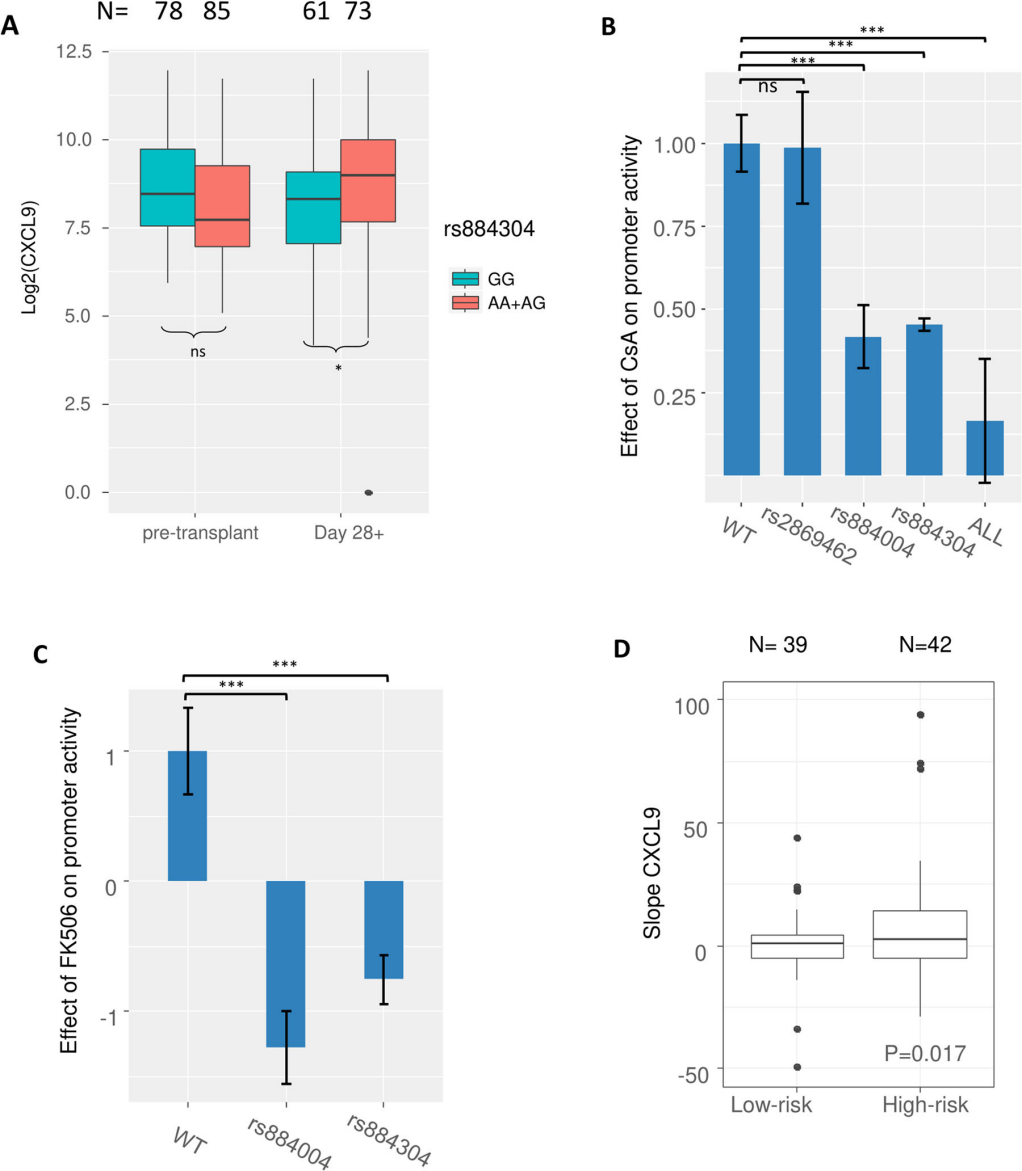
The association of rs884304 with IFN-γ induced promoter activity and CXCL9 recovery. **A** In patients, high-risk genotypes (AA + AG) of genetic risk group A (rs884304) were associated with significantly higher day +28 serum CXCL9 levels, but not with pre-transplant CXCL9. **B** In luciferase reporter assays, IFN-γ activation revealed that the constructs carrying variant allele of rs884304 (*P* < 0.001), rs884004 (*P* < 0.001), and the one carrying variant alleles of all the three SNPs (*P* = 0.001), but not rs2869462, were associated with significantly reduced suppressive effect of CsA compared to the wild-type construct. The results were collected from three independent experiments. Luciferase activity was normalized to 1 relative to the WT and all data were plotted as the mean ± SEM. **C** Similar to (**B**), with FK506 (tacrolimus) as calcineurin inhibitor. Results were collected from three independent experiments. Luciferase activity was normalized to 1 relative to the WT and all data were plotted as the mean ± SEM. **D** Recovery rates of CXCL9 serum levels in patients from the lowest value post alloSCT until day 28+ in the presence of calcineurin inhibitors (slope CXCL9) was higher in the genetic high-risk group. n.s. not significant; *P ≤ 0.05; ***P < 0.001.

We hypothesized that *CXCL9* promoter activity is regulated by the polymorphism rs884304. Indeed, rs884304AA/AG correlated with increased CXCL9 serum levels on day +28 (Fig. 3A). We performed luciferase reporter assays in the presence and absence of IFN-γ and CNIs to mimic the in vivo environment of post-alloSCT recipients.

All five constructs with the insert fragment possessed *CXCL9* promoter activity and showed strong responses to IFN-γ treatment. On average, a 9.7-fold increase of the luciferase activity was observed for the five constructs upon IFN-γ activation, while the empty vector showed no response (Supplemental Fig. 8).

Upon IFN-γ activation, significantly reduced suppressive effects of CsA on the luciferase activity $\left({\mathrm{Effect}}\; {\mathrm{of}}\; {\mathrm{CsA}} = \left(1 -\frac{{\mathrm{{luci}}\;{\mathrm{fearse}}}\; {\mathrm{activity}}\; {\mathrm{with}}\; {\mathrm{CsA}}}{{\mathrm{{luci}}\;{\mathrm{fearse}}}\; {\mathrm{activity}}\; {\mathrm{without}}\; {\mathrm{CsA}}}\right)^\ast 100\%\right)$ in the constructs carrying variant alleles of rs884304 (*P* < 0.001), rs884004 (*P* < 0.001), and the construct with all three SNPs (*P* = 0.001) were observed compared to the wild-type construct, while the construct carrying the variant allele of rs2869462 showed no significant difference (Fig. 3B). Similar effects of the rs884304 (*P* < 0.001) and rs884004 (*P* < 0.001) variant alleles were observed for FK506 (Fig. 3C).

CXCL9 serum levels decreased within 2 weeks after alloSCT and normalized until day +28 (Supplemental Fig. 9). CXCL9 serum level kinetics was modelled by slope calculation. Slope CXCL9 represents the CXCL9 recovery rate from the lowest point between days 0 and +14 until day +28 post transplant.

Patients carrying the variant allele A of rs884304 had a significantly steeper slope CXCL9 (Fig. 3D; *P* = 0.017) in the training cohort. The steeper slopes suggest that CXCL9 levels increased more rapidly during hematological reconstitution in the context of CNI.

## Discussion

GVHD remains a serious cause of morbidity and mortality in alloSCT. cGVHD is associated with poor quality of life, and prediction and prevention of cGVHD remain difficult. In the current study, we established a genetic risk group-based *CXCR3* ligand genes of the recipients as a novel predictor of severe cGVHD after alloSCT. Knowledge of the recipients’ genetic risk could thus trigger altered immunosuppressive or immunomodulating regimens, including ATG or SEP prophylaxis.

For cGVHD prediction, most studies focused on diagnostic and prognostic biomarkers at disease onset or in established cGVHD. A biomarker panel including CXCL9 at day +100 has been developed that can predict cGVHD^31^. However, the pathogenesis of cGVHD initiates earlier^32,33^. In the present study, CXCL9 serum levels on day +28 were associated with the risk of severe cGVHD. These data suggest that immune-mediated pathways activated early after alloSCT may contribute to subsequent development of severe cGVHD.

In luciferase reporter assays, the variant allele of rs884304 and rs884004 showed resistance to the immunosuppressive effect of CNIs upon IFN-γ activation. Calcineurin has been shown to interact with transcription factors including increasing NFIC transactivation and enhancing MEF2 DNA binding^34–36^. Chromatin immunoprecipitation-sequencing peaks of transcription factor binding sites for NIFC and MEF2A are found in the region containing the SNP rs884304 in ENCODE (Encyclopedia of DNA Elements) data^37^. It can be hypothesized that these enhancer risk variants can affect the binding of NIFC or MEF2, leading to less interaction with calcineurin, and thus reduced suppressive effects of CNIs. This may partly explain the effect of the CXCR3 ligands’ SNPs on CXCL9 expression and severe cGVHD. The effects of the other three SNPs and how the combined effect alters CXCL9 expression need to be further investigated.

The CXCR3 signalling pathway could play a role in the pathogenesis of cGVHD by driving donor effector T cells into target tissues of the recipient^38^. Moreover, CXCR3 regulates apoptotic death of endothelial cells, and high concentrations of CXCL9 induced breakdown of the endothelial barrier through the CXCR3 pathway^20,39^. The interaction between the CXCR3 system and endothelial cells may facilitate the trafficking of donor T cells into target tissues and aggregate inflammation and tissue damage in cGVHD patients. This endothelial side of CXCR3 signalling may explain why SEP also reduced severe cGVHD in patients with already high CXCL9 levels on day +28.

B cells are recognized as important players in the pathophysiology of cGVHD^40,41^. Notably, expression of CXCR3 is associated with aberrant B cell subpopulations reported in cGVHD^19^ and in other autoimmune diseases^42–44^.

The SEP cohort in Heidelberg University Hospital showed a lower incidence of TAM, and reduced NRM in patients with a high risk of endothelial dysfunction^45–47^. UDCA has been shown to reduce early NRM and severe acute GVHD, but not late NRM nor cGVHD^48^. CXCL9 is an IFN response gene, and statins were shown to inhibit type I and type II IFNs and IFN responses^49–52^. Statins were reported to reduce cGVHD in mice^53^ and in CsA-treated patients^54^. However, evaluating the individual contributions of UDCA and statins to the protective effect is not possible in our cohort. In the current study, SEP was associated with reduced day +28 serum levels of CXCL9 and a lower incidence of severe cGVHD in high-risk patients. In addition, the risk of severe cGVHD was also lower in SEP-treated patients with high day +28 CXCL9. These results suggest that the favorable outcome of SEP on preventing severe cGVHD may be a result of the combined effects of immune modulation and endothelial protection. Nonetheless, the exact mechanism of how SEP may be able to protect patients from severe cGVHD is still elusive.

ATG is usually given from days −3 to −1 before alloSCT^55^. ATG significantly reduced the risk of severe cGVHD, but this effect was not different between high- and low-risk groups. This suggests that ATG amended the effects of high day +28 CXCL9 on the incidence of cGVHD, rather than reducing the serum cytokine levels. All unrelated donors including mismatched donors received ATG in our study; therefore, we did not find any impact of HLA matching on severe cGVHD in multivariable models.

The main limitation of our study is represented by its retrospective and observational design making the findings vulnerable to unavoidable bias and unknown confounders. However, compared to published analyses on cGVHD-associated SNPs, our study includes an independent validation cohort, and suggests functional consequences of the SNPs on protein expression in the context of impeded suppressive effects by CNI. Knowing that the CXCL9 SNP status may improve patient management by promoting the use of endothelial protection strategies in high-risk patients.

In conclusion, the risk of severe cGVHD could be predicted by the genetic risk based on CXCR3 ligand genes in the absence of SEP. SEP may reduce the risk of severe cGVHD by regulating serum CXCL9 levels, and thus warrants further study.

## Supplementary information

supplemental Figures

supplemental materials
