# Supplementary material for: Polymorphisms in CXCR3 ligands predict early CXCL9 recovery and severe chronic GVHD
Source: Blood Cancer J. 2021 Feb 27;11(2):42. doi: 10.1038/s41408-021-00434-2 (PMC7914250; doi:10.1038/s41408-021-00434-2)

Suppl. Figure 1

CXCL4, CXCL4V1 and CXCL9-11

CXCR3

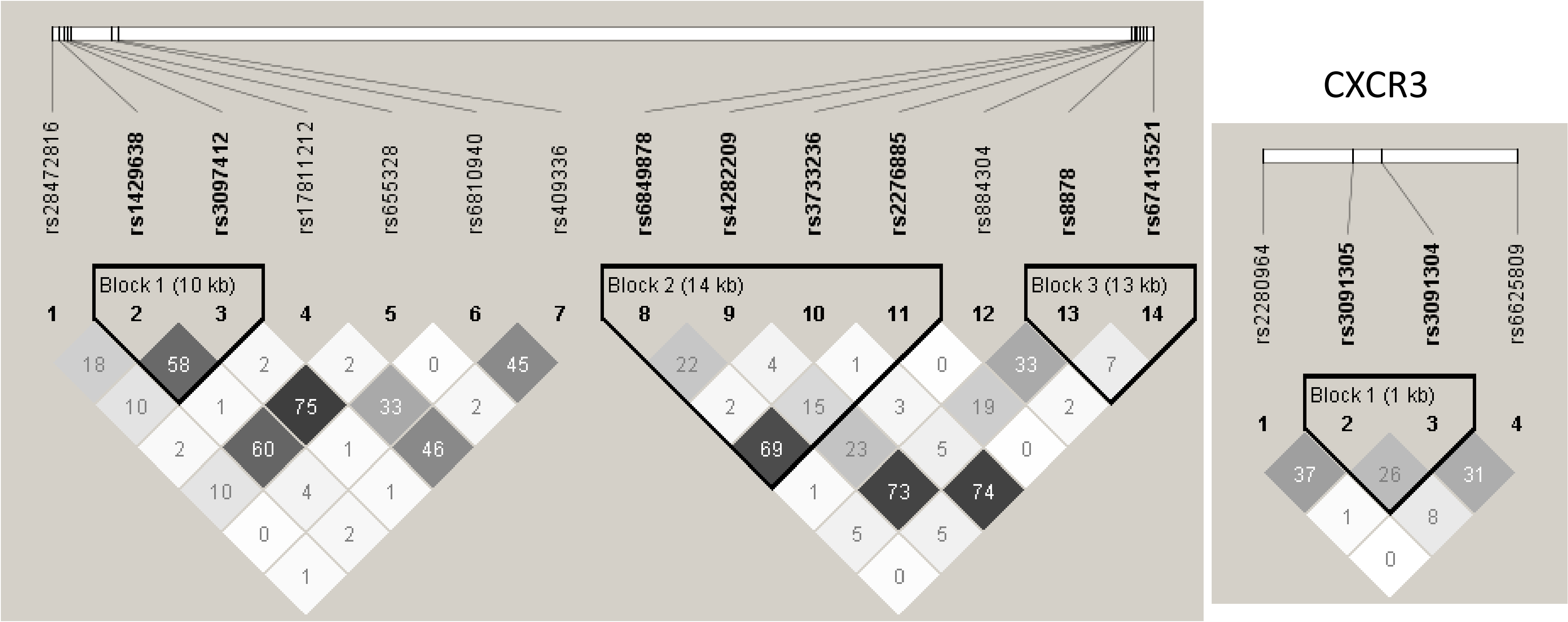

Suppl. Figure 2

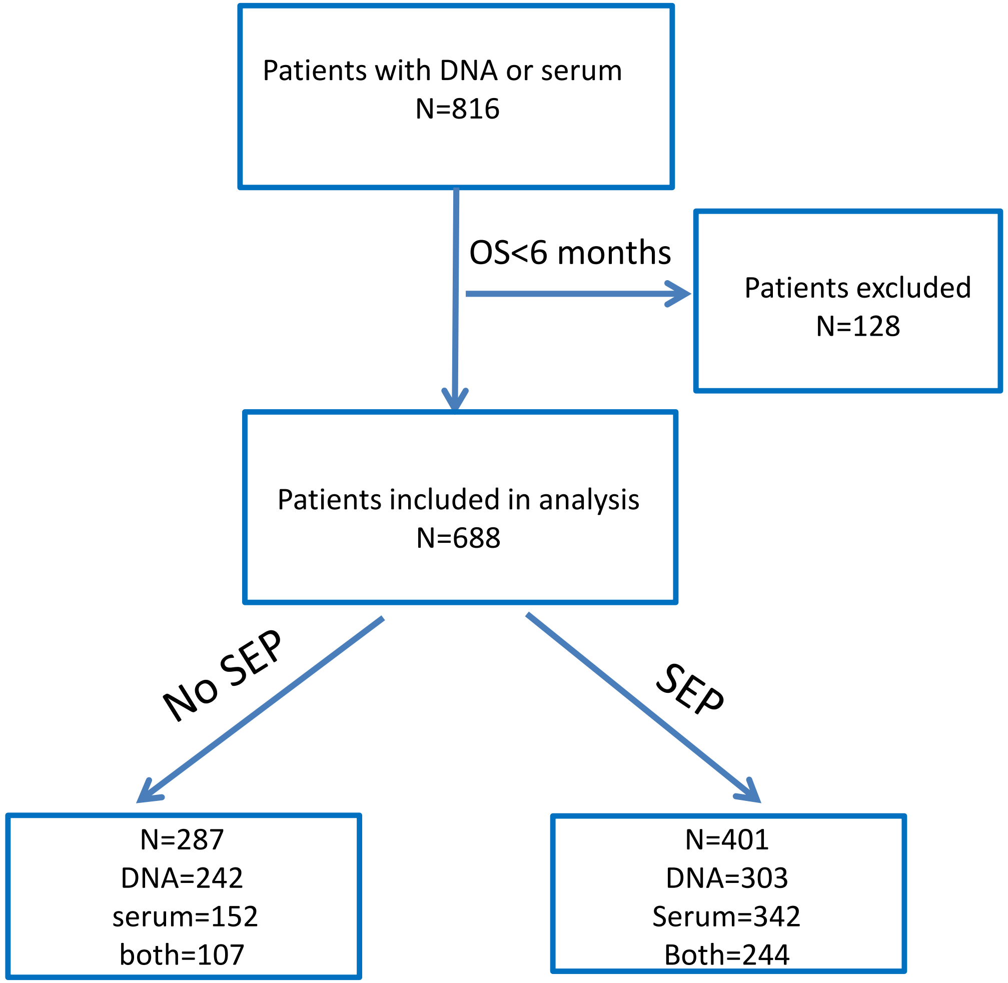

Abbreviations:
OS: Overall Survival (month); SEP: statin-based endothelial protection.

Suppl. Figure 3

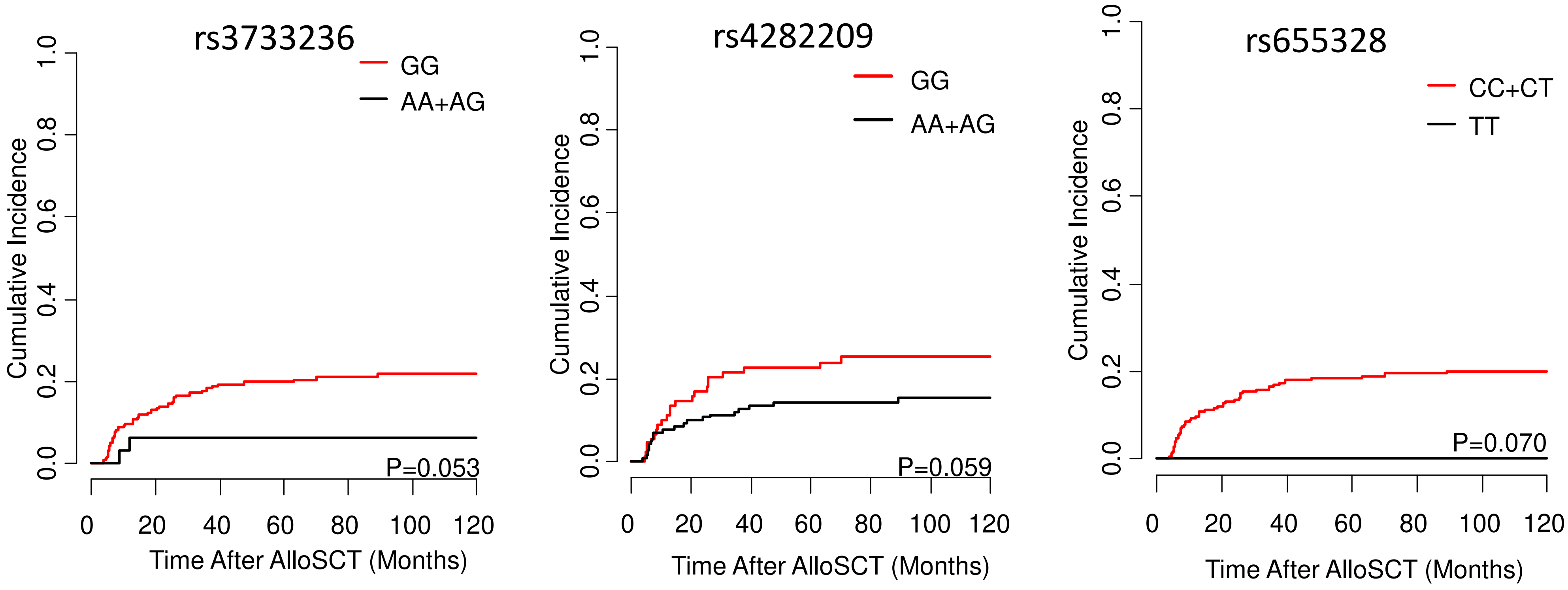

GG	201	119	91	87	79	48	33
AA+AG	36	24	19	15	15	12	5

GG	93	57	39	35	32	17	9
AA+AG	148	86	71	67	62	42	29

CC+CT	224	133	101	95	88	55	35
TT	13	10	9	7	6	5	3

Suppl. Figure 4

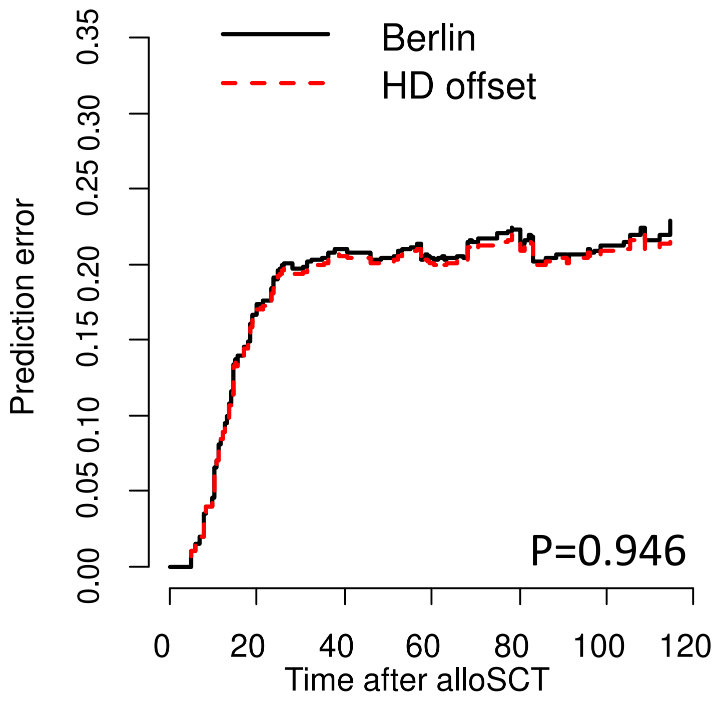

Suppl. Figure 5A

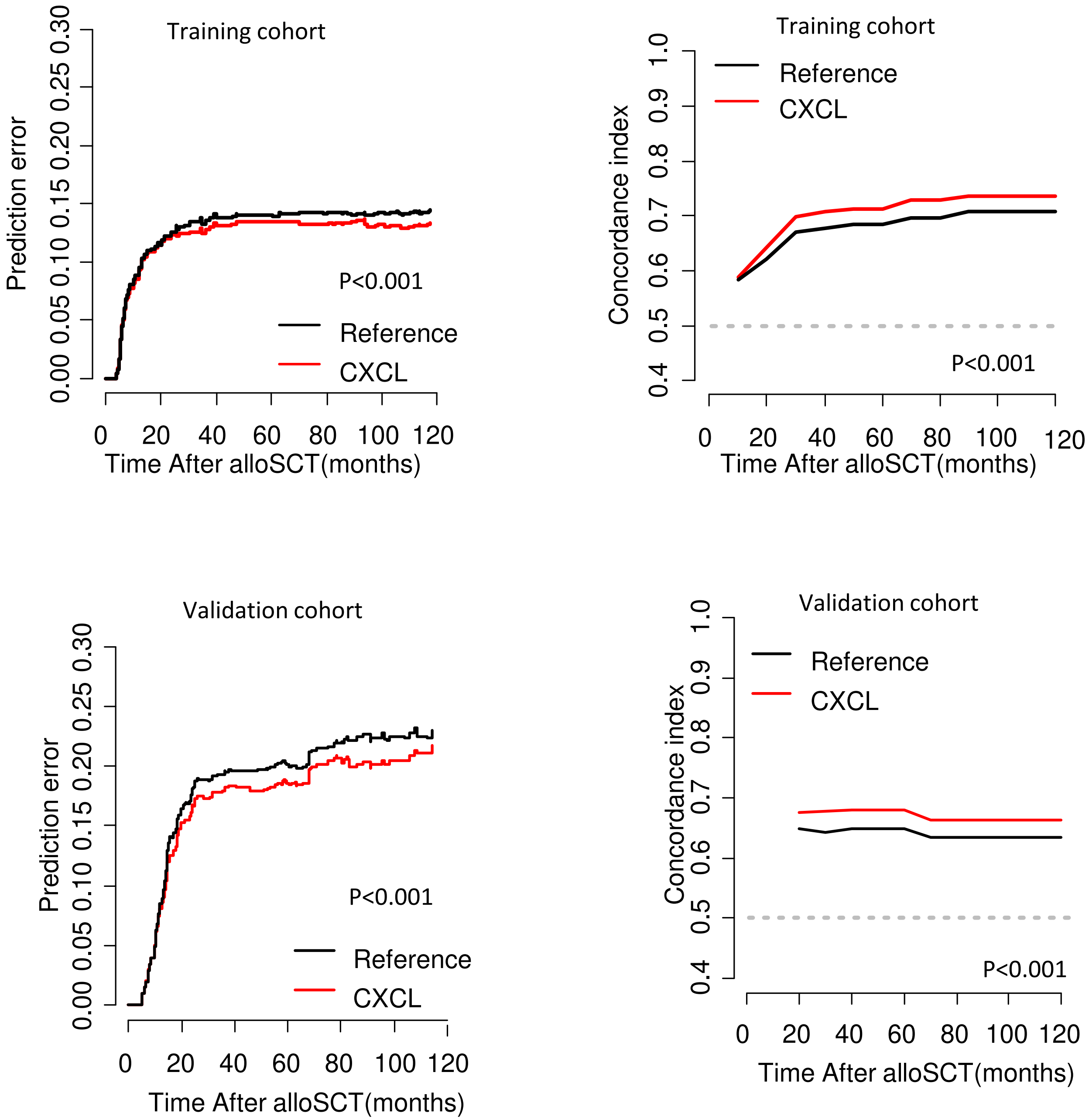

Suppl. Figure 5B

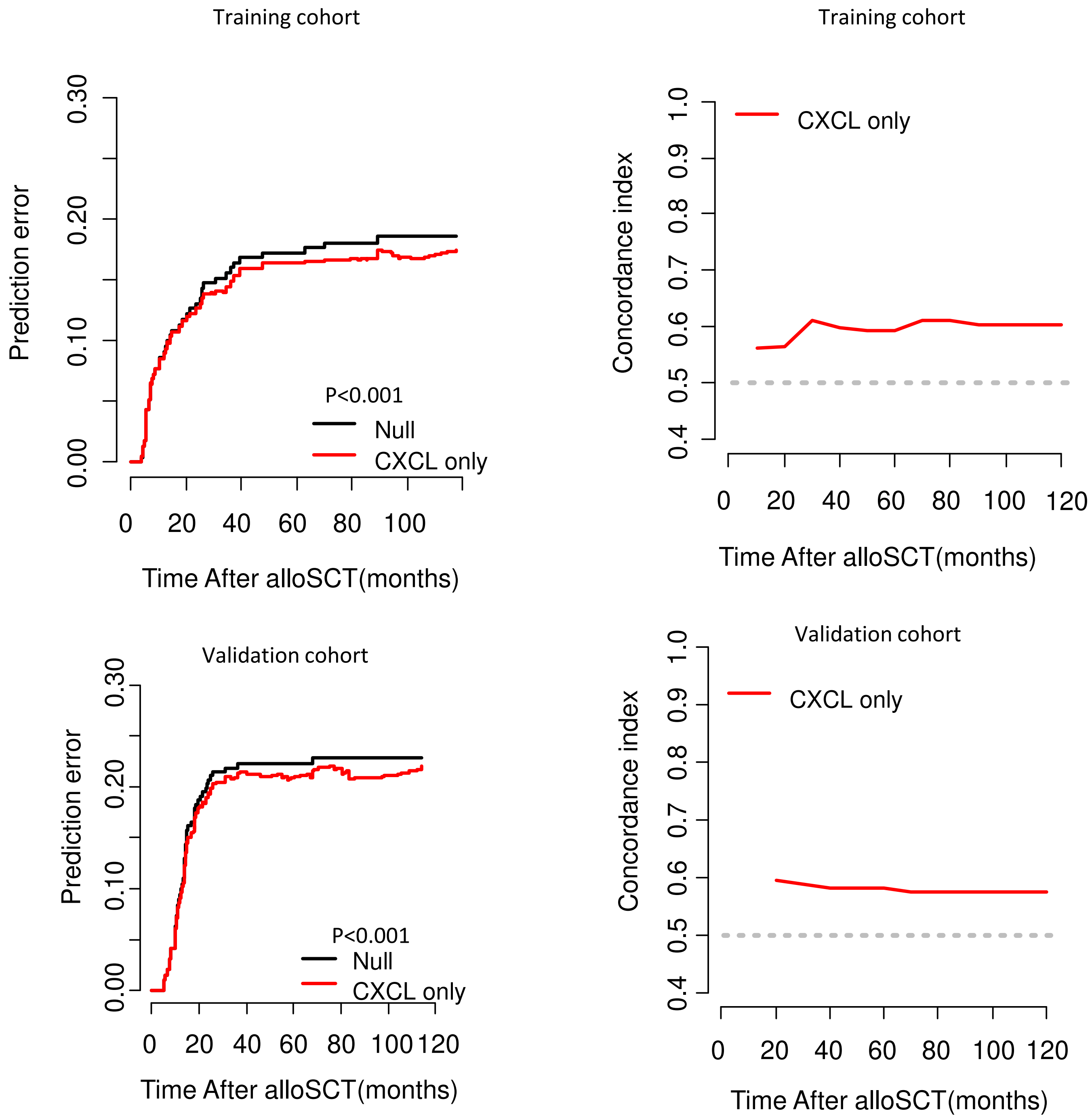

Suppl. Figure 6

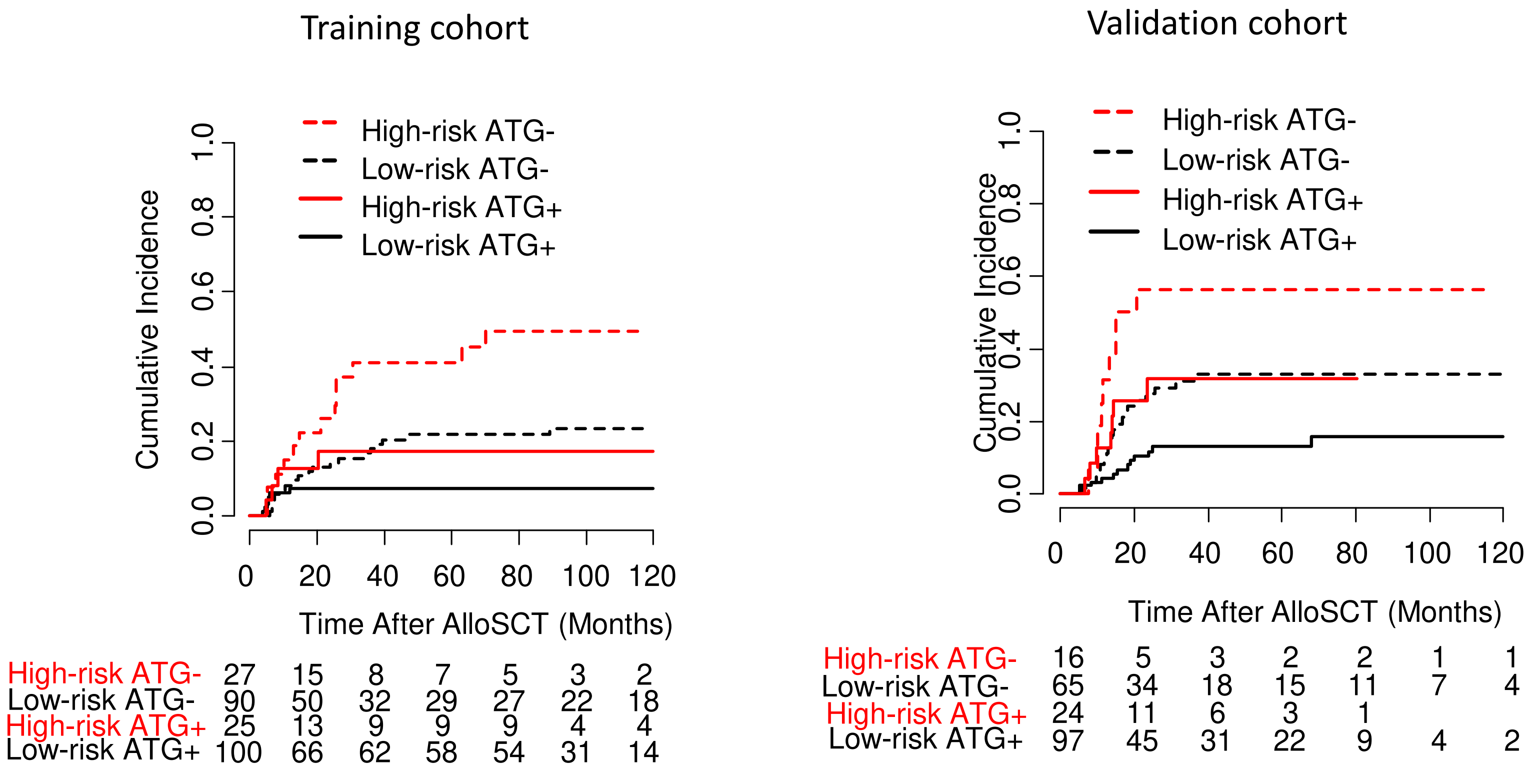

Suppl. Figure 7

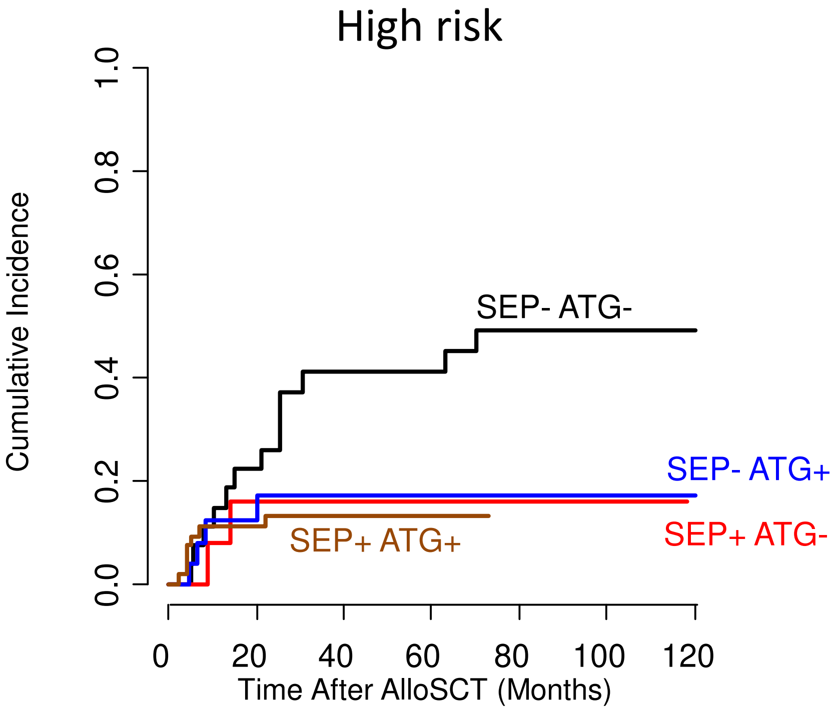

	0	20	40	60	80	100	120
SEP- ATG-	27	15	8	7	5	3	2
SEP+ ATG-	14	6	4	2	1	1	
SEP- ATG+	25	13	9	9	9	4	4
SEP+ ATG+	54	30	19	4			

5 year cumulative incidence: (95%CI)

Statin- ATG-: 41.1% (25.2%-61.9%)

Statin+ ATG-: 16.1% (4.3%-50.1%)

Statin- ATG+: 17.1% (6.8%-39.6%)

Statin+ ATG+: 13.2% (6.5%-25.7%)

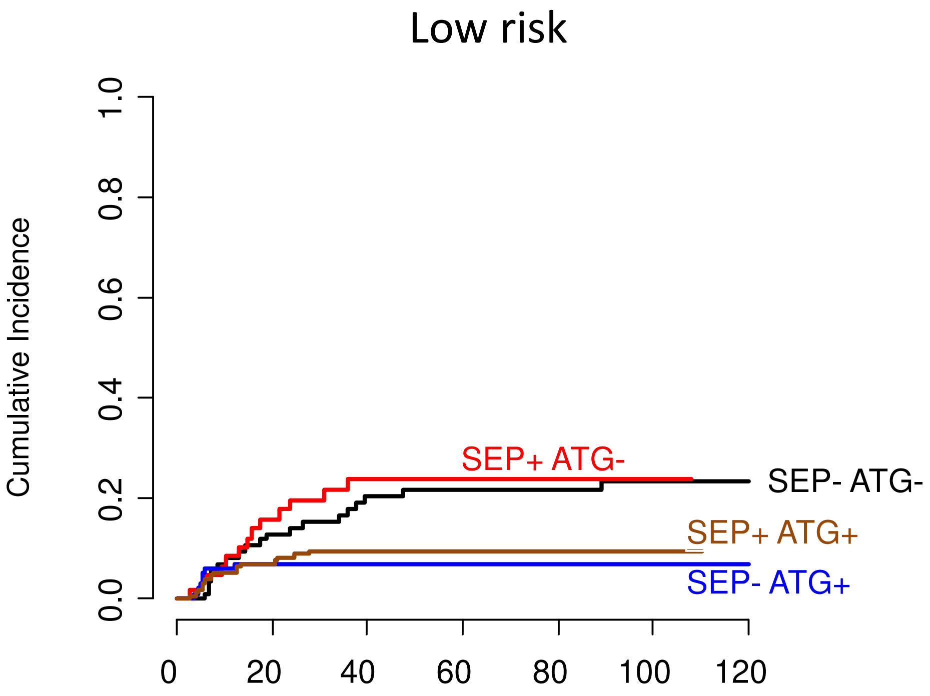

	0	20	40	60	80	100	120
SEP- ATG-	90	50	32	29	27	22	18
SEP+ ATG-	61	32	22	14	2	1	
SEP- ATG+	100	66	62	58	54	31	14
SEP+ ATG+	174	116	75	29	4	1	

5 year cumulative incidence: (95%CI)

Statin- ATG-: 20.4% (13.2%-30.7%)

Statin+ ATG-: 23.6% (14.4%-37.3%)

Statin- ATG+: 7.1% (3.4%-14.2%)

Statin+ ATG+: 9.5% (5.9%-15.1%)

Suppl. Figure 8

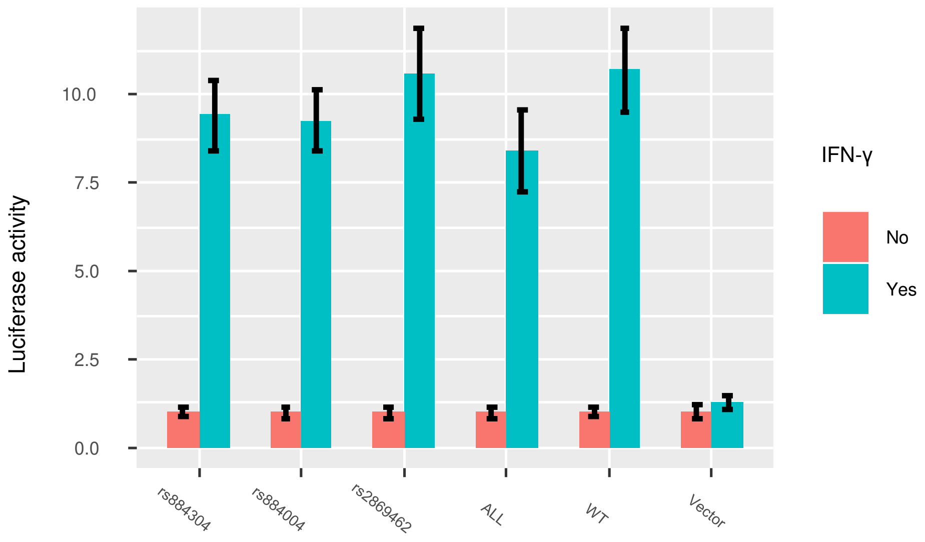

Suppl. Figure 9

Training cohort

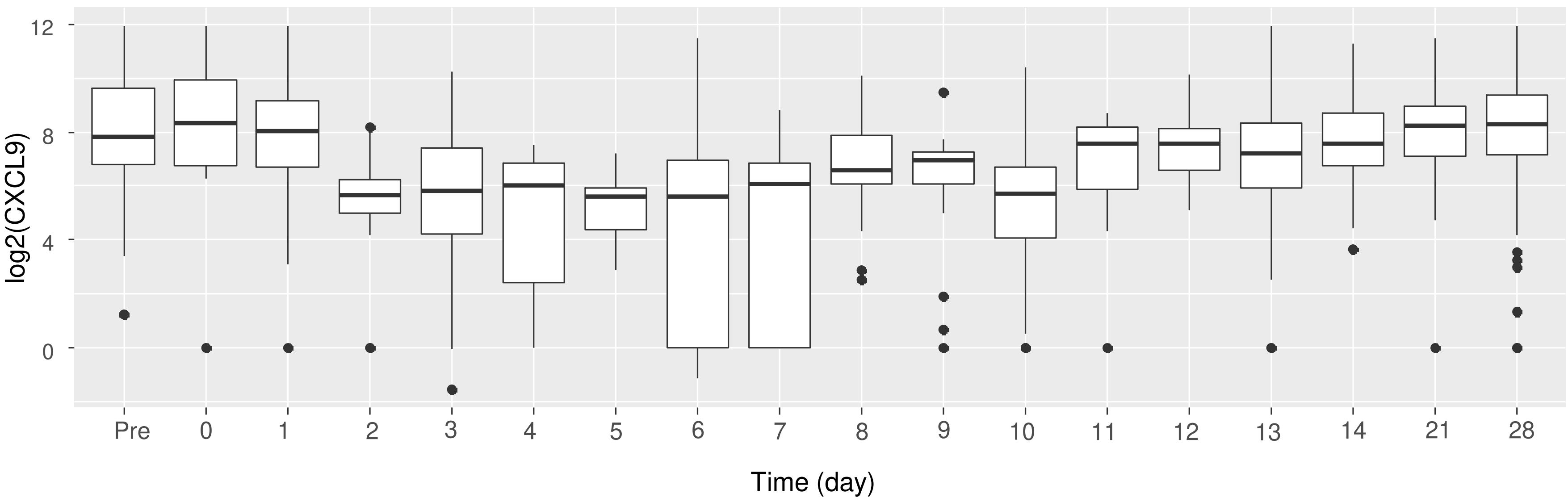

SEP cohort

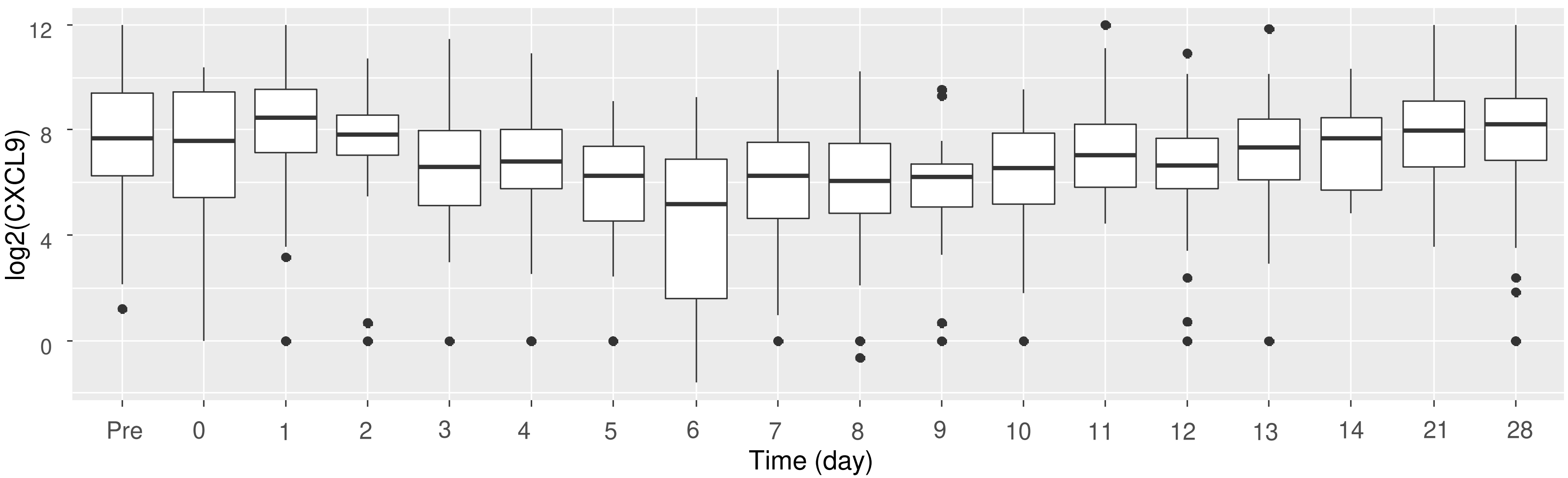

Supplement: Supplementary file 1 — supplemental Figures [file 41408_2021_434_MOESM1_ESM.pdf]
