# Supplementary material for: Polymorphisms in CXCR3 ligands predict early CXCL9 recovery and severe chronic GVHD
Source: Blood Cancer J. 2021 Feb 27;11(2):42. doi: 10.1038/s41408-021-00434-2 (PMC7914250; doi:10.1038/s41408-021-00434-2)
Supplement: Supplementary file 2 — supplemental materials [file 41408_2021_434_MOESM2_ESM.pdf]

Supplemental Figure Legends

Suppl.Fig.1. linkage disequilibrium maps of the Single nucleotide polymorphisms (SNPs) from CXCL4 (v1), CXCL9-11 and CXCR3.

Suppl.Fig.2. Flowchart of study cohort in Heidelberg.

Suppl.Fig.3. The effects of the SNPs in genetic risk group B on severe chronic GVHD in the traning cohort.

Suppl.Fig.4. Validation of the genetic risk group effect using prediction error curves.

The superimposition of the prediction error curves for the two different cox models suggest that the effect of genetic risk group from training cohort (HD offset) is similar with that from validation cohort (Berlin), i.e., the effect could be considered validated. For detailed description of the validation method see "Supplemental Statistical Methods" in the APPENDIX.

Suppl.Fig.5. Assessment of the genetic risk group effect using prediction error curves.

A): The prediction error and concordance index curves of multivariable models with (CXCL) and without (Reference) genetic risk group predictor in the training cohort and validation cohort. P values were calculated by Wilcoxon rank sum test.

B): The prediction error and concordance index curves of univariable model of the genetic risk group predictor in the training cohort and validation cohort. 'Null' model is based on Kaplan-Meier estimator only. P values were calculated by Wilcoxon rank sum test.

Suppl.Fig.6. Anti-thymocyte globulin (ATG) associated with significantly reduced risk of severe GVHD in the training cohort and validation cohort.

Suppl.Fig.7. The effects of statin-based protection (SEP) and Anti-thymocyte globulin (ATG) in patients from Heidelberg cohort stratified by genetic risk.

Suppl.Fig.8. Confirmation of IFN-γ induced CXCL9 promoter activity for all the five constructs.

In the presence of IFN-γ treatment, all five constructs showed significantly increased Luciferase activity compared to control experiments without IFN-γ. The empty vector showed no significant activity. Luciferase activity was normalized to 1 relative to the respective construct without IFN-γ treatment and all data were plotted as the mean ± SEM.

Suppl.Fig.9. Time course of serum CXCL9 concentrations in the early period after alloSCT

The trend of dynamic changes of serum CXCL9 levels until day +28 post-alloSCT in training and SEP cohort revealed a nadir around day 5-7 and a recovery thereafter.

APPENDIX

Supplemental Statistical methods

Validation of the genetic risk group

To confirm and validate the effect of the CXCR3 ligands genetic risk group on the risk of developing severe chronic GVHD, the cause-specific Cox model was fitted to the validation data set, including an offset that was equal to the effect of the genetic risk group in the model on the basis of patients from the training cohort. That means that the effect of the genetic risk group was transferred to the model for the validation cohort. The effect of the genetic risk group on severe chronic GVHD was estimated in addition to this transferred effect to verify whether the effect in the validation cohort differed from what had been observed in the training cohort.

Two cause-specific Cox proportional hazards models for severe chronic GVHD were evaluated using prediction error curves. The effect of combined genetic risk group was either transferred from the multivariate model based on the training set or it was re-estimated based on the data of the validation cohort. Both models included the same covariates age, diagnosis (lymphoid vs. myeloid), matched vs. mismatched donor, sex of donor and recipient and use of ATG (**Suppl. Figure 4**).

The 632+ bootstrap was applied for the estimation of the prediction error.[1] 1000 bootstrap subsamples were drawn from the validation data set and the two models explained above were fitted in each subsample. The resulting predictive model was then fitted to the out-of-bag sample (patients not contained in the respective subsample) to estimate the time-dependent prediction error for all event times in the validation data set. Prediction error was estimated using Brier score and calculated as weighted combination of the apparent estimate and the bootstrap estimate.[2,3]

1. Efron B, Tibshirani R: Improvements on Cross-Validation: The 632+ Bootstrap Method. Journal of the American Statistical Association 92:548-560, 1997
2. Gerds TA, Schumacher M: Consistent Estimation of the Expected Brier Score in General Survival Models with Right-Censored Event Times. Biometrical Journal 48:1029-1040, 2006
3. Mogensen UB, Ishwaran H, Gerds TA: Evaluating Random Forests for Survival Analysis Using Prediction Error Curves. Journal of Statistical Software 50, 2012

Suppl. table 1. Patient characteristics in training and validation cohort separated by genetic risk groups.

	genetic high risk** training no SEP n=52	genetic low risk* Training no SEP n=190	p-value	genetic high risk** validation n=40	genetic low risk* validation n=162	p-value	genetic high risk** SEP n= 68	genetic low risk* SEP n=235	p-value
**Median age at alloSCT** (years, range)	50 (18-68)	50 (17-71)	0.81	52 (20-69)	49 (19-72)	0.41	57 (22-76)	56 (19-74)	0.50
**Sex** (n, %)									
Female	24 (46)	69 (36)	0.20	12 (30)	60 (37)	0.46	24 (35)	95 (40)	0.48
Male	28 (54)	121 (64)		28 (70)	102 (63)		44 (65)	140 (60)	
**Donor**									
RD	23 (44)	66 (35)	0.26	9 (14)	56 (35)	0.19	15 (22)	65 (28)	0.44
UD	29 (56)	124 (65)		31 (86)	106 (65)		53 (78)	170 (72)	
**Donor-recipient HLA matching**									
Matched donor	43 (83)	136 (72)	0.11	37 (93)	158 (98)	0.14	62 (91)	186 (79)	0.03
Mismatched donor	9 (17)	54 (28)		3 (7)	4 (2)		6 (9)	49 (21)	
**Sex mismatch (Donor-Recipient)** (n, %)									
Male-Male, Female-Female	26 (47)	117 (62)	0.30	20 (50)	89 (55)	0.77	44 (65)	126 (54)	0.27
Male-Female	16 (33)	43 (22)		9 (23)	36 (22)		14 (20)	60 (25)	
Female-Male	10 (20)	30 (16)		11 (27)	37 (23)		10 (15)	49 (21)	
**Disease** (n, %)									
AML	13 (25)	48 (25)	0.18	17 (42)	66 (41)	0.56	20 (30)	84 (36)	0.67
MDS, MPN, AA	14 (27)	27 (17)		11 (28)	33 (20)		13 (19)	40 (17)	
Lymphoma, CLL	18 (35)	77 (44)		11 (28)	50 (31)		24 (35)	84 (36)	
MM, Amyloidosis	7 (13)	38 (20)		1 (2)	13 (8)		11 (16)	27 (11)	
**Disease Score before alloSCT** (n, %)									
0	19 (36)	60 (32)	0.08	13 (32)	79 (49)	0.01	27 (40)	75 (32)	0.53
1	4 (8)	29 (15)		15 (38)	65 (40)		25 (37)	88 (37)	
2	24 (46)	96 (51)		9 (23)	16 (10)		14 (20)	66 (28)	
NA	5 (10)	5 (3)		3 (7)	2 (1)		2 (3)	6 (3)	
**Stem cell source** (n, %)									
Peripheral stem cells	48 (92)	182 (96)	0.29	40 (100)	160 (99)	1.00	67 (99)	220 (94)	0.13

Bone marrow stem cells	4 (8)	8 (4)		0 (0)	2 (1)		1 (1)	15 (6)	
**Conditioning** (n, %)									
MAC	9 (17)	36 (19)	0.54	16 (40)	58 (36)	0.71	2 (3)	9 (4)	0.28
APL	0 (0)	6 (3)					4 (6)	30 (13)	
RIC	43 (83)	148 (78)		24 (60)	104 (64)		62 (91)	195 (83)	
**ATG** (n, %)									
no	27 (52)	90 (47)	0.64	16 (40)	65 (40)	1.00	14 (21)	61 (26)	0.43
yes	25 (48)	100 (53)		24 (60)	97 (60)		54 (79)	174 (74)	

P-values were calculated using the Mann Whitney U-Test (age), Fisher's exact test.

*__Low risk:__ rs884304GG, rs3733236AA/AG, rs4282209AA/AG, rs2276885GG, rs655328TT, rs3097412TT.

High risk:** no low risk genotype.

Abbreviations:

AA: aplastic anemia; ALL: acute lymphoblastic leukemia; alloSCT: allogeneic stem cell transplantation; AML: acute myelogenous leukemia; ATG: anti-thymocyte globulin; CLL: chronic lymphocytic leukemia; Disease score: 0=CR1, 1=CR2, 2=all other; MAC: myeloablative conditioning; MPS: myeloproliferative syndrome; MM: multiple myeloma; RD: related donor; RIC: reduced intensity conditioning; UD: unrelated donor.

Suppl. table 2. Genotype distributions and effects of single-nucleotide polymorphisms in the training cohort (no SEP)

genotypes	patients	events[a]	HR (95%CI)[b]	p[c]	genotypes	patients	events[a]	HR (95%CI)[b]	p[c]
rs884304 (CXCL9)					rs2276885 (CXCL9)				
GG	117	13			AA	144	26		
AG	97	21	2.08 (1.04-4.15)	0.04	AG	80	16	1.11 (0.60-2.08)	0.73
AA	27	9	3.25 (1.39-7.60)	0.01	GG	11	1	0.40 (0.05-2.93)	0.36
AA+AG vs. GG			2.33 (1.21-4.46)	0.01	GG+AG vs. AA			1.01 (0.55-1.86)	0.98
AA vs. AG+GG			2.02 (0.97-4.21)	0.06	GG vs. AG+AA			0.33 (0.05-2.43)	0.28
rs3733236 (CXCL9)					rs6849878 (CXCL9)				
GG	201	41			GG	132	26		
AG	33	2	0.28 (0.07-1.16)	0.08	AG	81	13	0.78 (0.40-1.52)	0.47
AA	3	0	N/A	N/A	AA	23	4	0.90 (0.31-2.57)	0.84
AA+AG vs. GG			0.25 (0.06-1.04)	0.06	AA+AG vs. GG			0.81 (0.44-1.49)	0.49
AA vs. AG+GG			N/A	N/A	AA vs. AG+GG			0.93 (0.33-2.59)	0.88
rs4282209 (CXCL9)					rs8878 (CXCL10)				
GG	93	22			GG	75	18		
AG	119	16	0.56 (0.29-1.06)	0.08	AG	129	19	0.62 (0.24-1.75)	0.14
AA	29	5	0.71 (0.27-1.87)	0.49	AA	34	5	0.65 (0.24-1.75)	0.39
AA+AG vs. GG			0.59 (0.32-1.07)	0.08	AA+AG vs. GG			0.62 (0.34-1.15)	0.13
AA vs. AG+GG			0.95 (0.37-2.40)	0.90	AA vs. AG+GG			0.84 (0.33-2.13)	0.72

rs67413521 (CXCL11)					rs655328 (CXCL4V1)				
AA	190	36			CC	125	23		
AG	35	6	0.67 (0.33-1.33)	0.25	CT	99	19	1.01 (0.55-1.85)	0.99
GG	3	0	N/A	N/A	TT	13	0	N/A	N/A
GG+AG vs. AA			0.72 (0.38-1.37)	0.32	TT+CT vs. CC			0.87 (0.48-1.60)	0.66
GG vs. AG+AA			N/A	N/A	TT vs. CT+CC			N/A	N/A
rs3097412 (CXCL4V1)					rs17811212 (CXCL4V1)				
GG	122	25			GG	210	36		
GT	100	18	0.82 (0.45-1.50)	0.52	AG	30	7	1.54 (0.69-3.47)	0.29
TT	15	0	N/A	N/A	AA	0	0	N/A	N/A
TT+GT vs. GG			0.70 (0.38-1.28)	0.24	AA+AG vs. GG			1.54 (0.69-3.47)	0.29
TT vs. GT+GG			N/A	N/A	AA vs. AG+GG			N/A	N/A
rs1429638 (CXCL4V1)					rs28472816 (CXCL4V1)				
CC	166	33			TT	172	34		
AC	68	10	0.74 (0.37-1.51)	0.41	CT	57	8	0.67 (0.31-1.45)	0.31
AA	5	0	N/A	N/A	CC	7	1	0.74 (0.10-5.42)	0.77
AA+AC vs. CC			0.68 (0.34-1.39)	0.29	CC+CT vs. TT			0.68 (0.33-1.42)	0.30
AA vs. AC+CC			N/A	N/A	CC vs. CT+TT			0.81 (0.11-5.89)	0.84
rs409336 (CXCL4)					rs6810940 (CXCL4)				
AA	185	33			TT	159	28		
AC	48	10	1.23 (0.61-2.51)	0.56	CT	71	14	1.07 (0.56-2.04)	0.83
CC	3	0	N/A	N/A	CC	9	1	0.59 (0.08-4.35)	0.61
CC+AC vs. AA			1.14 (0.56-2.30)	0.73	CC+CT vs. TT			1.02 (0.54-1.91)	0.96
CC vs. AC+AA			N/A	N/A	CC vs. CT+TT			0.58 (0.08-4.21)	0.59

rs2280964 (CXCR3)					rs6625809 (CXCR3)				
CC	171	36			GG	213	38		
CT	27	3	0.60 (0.18-1.95)	0.39	GT	13	2	1.08 (0.26-4.50)	0.91
TT	39	4	0.50 (0.18-1.41)	0.19	TT	8	2	1.96 (0.47-4.50)	0.36
TT+CT vs. CC			0.54 (0.24-1.21)	0.13	TT+GT vs. GG			1.40 (0.50-3.92)	0.53
TT vs. CT+CC			0.53 (0.19-1.48)	0.22	TT vs. GT+GG			1.95 (0.47-8.07)	0.36
rs3091304 (CXCR3)					rs3091305 (CXCR3)				
TT	190	33			TT	128	26		
CT	19	4	1.42 (0.50-4.01)	0.51	GT	27	3	0.60 (0.18-1.97)	0.39
CC	29	5	0.98 (0.68-2.51)	0.96	GG	82	13	0.79 (0.41-1.54)	0.49
CC+CT vs. TT			1.34 (0.54-2.37)	0.74	GG+GT vs. TT			0.75 (0.40-1.39)	0.35
CC vs. CT+TT			0.95 (0.37-2.41)	0.91	GG vs. GT+TT			0.85 (0.44-1.63)	0.62

a: Number of patients who developed severe chronic GVHD.

b: Hazard ratio and 95% confidence interval from additive, dominant or recessive using cause-specific cox regression for the outcome of severe chronic GVHD. Death without severe chronic GVHD was the competing event for severe chronic GVHD. For a SNP with a major allele A and a minor allele a, the recessive model compares genotype aa with genotypes AA and Aa, AA and Aa used as the reference. The dominant model compares genotypes Aa and aa against the genotype AA, AA used as the reference. Additive model assumes that aa has a 2-fold increase in effect size compared to Aa, using AA as reference.

c: p-value from cause-specific cox regression for the outcome of severe chronic GVHD. Death without severe chronic GVHD was the competing event for severe chronic GVHD.

Abbreviations:

CI: confidence interval; HR: hazard ratio; N/A: not applicable.

Supplemental Table 3. Multivariable analysis of predictors of incidence of severe chronic graft-versus-host disease (GVHD), overall survival (OS), non-relapse mortality (NRM) and relapse in the SEP cohort (SEP, OS>=6 months, n=303)

	Severe chronic GVHD[a]				***OS***				***NRM[a]***		***Relapse[a]***	
**Covariates**	**N**	**ScGVHD**	**HR (95% CI)**	***P***	**N**	**death**	**HR (95% CI)**	***P***	**HR (95% CI)**	***P***	**HR (95% CI)**	***P***
**genetic risk**												
Low-risk group	235	29	Ref		235	77	Ref		Ref		Ref	
High-risk group	68	9	1.48(0.68-3.21)	0.321	68	25	1.15(0.72-1.82)	0.561	1.01(0.45-2.24)	0.985	1.34(0.80-2.23)	0.266
**Age**	303	38	0.97(0.95-1.00)	0.051	303	102	1.02(1.00-1.04)	0.074	1.04(1.01-1.08)	0.018	1.00(0.98-1.02)	0.966
**Recipient Sex**												
Female	119	14	Ref		119	35	Ref		Ref		Ref	
Male	184	24	1.06 (0.54-2.10)	0.866	184	67	1.18(0.78-1.79)	0.429	1.48(0.73-3.00)	0.282	0.97(0.61-1.54)	0.896
**Donor Sex**												
Female	104	13	Ref		104	26	Ref		Ref		Ref	
Male	199	25	1.26 (0.64-2.49)	0.508	199	76	1.64(1.04-2.58)	0.032	1.15(0.59-2.25)	0.686	1.74(1.04-2.91)	0.035
**Donor**												
Matched donor	248	30	Ref		248	80	Ref		Ref		Ref	
Mismatched donor	55	8	1.52(0.64-3.62)	0.345	55	22	1.55(0.93-2.58)	0.094	2.42(1.12-5.22)	0.025	1.12(0.60-2.07)	0.721
**Disease type**												
Myeloid[b]	157	20	Ref		157	51	Ref		Ref		Ref	
Lymphoid[c]	146	18	0.79(0.41-1.51)	0.473	146	51	1.09(0.73-1.63)	0.667	1.56(0.81-2.98)	0.182	0.96(0.61-1.51)	0.872
**ATG**												
No	75	15	Ref		75	29	Ref		Ref		Ref	
Yes	228	23	0.43(0.21-0.89)	0.022	228	73	0.71(0.45-1.13)	0.146	0.47(0.22-0.98)	0.045	0.70(0.41-1.17)	0.173

[a] HR and p-value from Cause-specific cox models for competing risk. Death without severe chronic GVHD was taken as the competing event for severe chronic GVHD. NRM and relapse were treated as competing events.

[b] Myeloid: acute myeloid leukemia, myelodysplastic and myeloproliferative syndromes.

[c] Lymphoid: acute lymphoblastic leukemia, chronic lymphocytic leukemia, T-/B-cell lymphoma and multiple myeloma.

Abbreviations:

ATG: anti-thymocyte globulin; CI: confidence interval; HR: hazard ratio; ScGVHD: severe chronic graft-versus-host disease.

Supplemental Table 4. Patient characteristics in the training, validation and statin-based endothelial protection (SEP) cohorts (Complete cohorts, including patients did not survive 6 months)

	**Training cohort no SEP n=343**	**Validation cohort no SEP n=237**	**SEP cohort n= 473**
**Median age at alloSCT** (years, range)	50 (17-71)	50 (19-72)	57 (19-76)
**Sex** (n, %)			
Female	137 (40)	85 (36)	182 (38)
Male	206 (60)	152 (64)	291 (62)
**Donor**			
RD	124 (36)	74 (31)	120 (25)
UD	219 (64)	163 (69)	353 (75)
**Donor-recipient HLA matching**			
Matched donor	252 (73)	227 (96)	378 (80)
Mismatched donor	91 (27)	10 (4)	95 (20)
**Sex mismatch (Donor-Recipient)** (n, %)			
Male-Male, Female-Female	194 (57)	128 (54)	262 (55)
Male-Female	87 (25)	55 (23)	116 (24)
Female-Male	62 (18)	54 (23)	95 (21)
**Disease** (n, %)			
AML	106 (29)	103 (43)	183 (38)
MDS, MPN, AA	59 (16)	46 (19)	80 (17)
Lymphoma, ALL, CLL	134 (37)	72 (30)	161 (34)
MM, Amyloidosis	67 (18)	16 (7)	49 (11)
**Disease Score before alloSCT** (n, %)			
0	110 (32)	110 (46)	155 (32)
1	52 (15)	88 (37)	166 (35)
2	165 (48)	34 (14)	143 (31)
NA	16 (5)	5 (3)	9 (2)
**Stem cell source** (n, %)			
Peripheral stem cells	320 (93)	235 (99)	448 (95)
Bone marrow stem cells	23 (7)	2 (1)	25 (5)
**Conditioning** (n, %)			
MAC	67 (20)	89 (38)	17 (4)

APL	10 (3)	0 (0)	65 (13)
RIC	266 (77)	148 (62)	389 (82)
NA			2 (1)
**ATG** (n, %)			
no	165 (48)	95 (40)	124 (26)
yes	178 (52)	142 (60)	349 (74)

a: measured pre-transplant.
b: measured at day+28 post-transplant.
Abbreviations:
AA: aplastic anemia; ALL: acute lymphoblastic leukemia; alloSCT: allogeneic stem cell transplantation; AML: acute myelogenous leukemia; ATG: anti-thymocyte globulin; CLL: chronic lymphocytic leukemia; Disease score: 0=CR1, 1=CR2, 2=all other; MAC: myeloablative conditioning; MPS: myeloproliferative syndrome; MM: multiple myeloma; NA: not available; RD: related donor; RIC: reduced intensity conditioning; UD: unrelated donor.

Supplemental Table 5. Effect of CXCL genetic risk group and CXCL9 on acute GVHD in the training cohort.

	acute GVHD	Grade 3-4 acute GVHD	NRM after GVHD
**Genetic risk group** High-risk vs. Low-risk	HR 0.76, 95%CI 0.48-1.19, P=0.225	HR 0.99, 95%CI 0.54-1.85, P=0.992	HR 1.36, 95%CI 0.65-2.86, P=0.418
**Genetic risk Group A** High-risk vs. Low-risk	HR 0.97, 95%CI 0.69-1.37, P=0.872	HR 1.28, 95%CI 0.77-2.14, P=0.347	HR 1.26, 95%CI 0.67-2.37, P=0.466
**Genetic risk Group B** High-risk vs. Low-risk	HR 0.83, 95%CI 0.56-1.23, P=0.346	HR 0.95, 95%CI 0.54-1.67, P=0.850	HR 1.46, 95%CI 0.75-2.84, P=0.269

[a] HR and p-value from Cause-specific Cox models for competing risks. Relapse and death without severe cGVHD were taken as competing events for severe cGVHD. NRM and relapse were treated as competing events.
